# EasyHybrid: An Interactive Graphical Environment for Quantum, Classical and Hybrid Simulations with pDynamo3

**DOI:** 10.1021/acs.jcim.5c02047

**Published:** 2025-11-11

**Authors:** Jose Fernando R. Bachega, Gustavo Hagen, Carlos Sequeiros-Borja, Kai Nikklas, Jorge Chahine, Luis Fernando M. S. Timmers, Martin J. Field

**Affiliations:** † Department of Pharmacosciences, Federal University of Health Sciences of Porto Alegre, 90050-170 Porto Alegre, RS, Brazil; ‡ Biotechnology Center, Graduate Program in Molecular and Cellular Biology, Federal University of Rio Grande do Sul, 90650-001 Porto Alegre, RS, Brazil; § Tunneling Group, Biotechnology Center, Krzywoustego 8, 44-100 Gliwice, Poland; ∥ 3D Development, deCode GmbH, 46145 Oberhausen, Germany; ⊥ Biological Structures Group, Multiuser Center for Biomolecular Innovation, São Paulo State University, São José do Rio Preto 15054-000, SP, Brazil; ○ Graduate Program in Biotechnology, University of Vale do Taquari, 95900-000 Lajeado, RS, Brazil; □ Laboratoire de Chimie et Biologie des Métaux, UMR5249, Université Grenoble I, CEA, CNRS, 38054 Grenoble, France; △ Theory Group, Institut Laue-Langevin, 38042 Grenoble, France

## Abstract

We present EasyHybrid, a free and open-source graphical interface for hybrid quantum chemical/molecular mechanical (QC/MM) simulations built on the pDynamo3 library. The software provides an intuitive environment for preparing, inspecting, and editing molecular systems, while supporting a broad range of simulations, including reaction coordinate scans, molecular dynamics, normal-mode analysis, Nudged Elastic Band, and umbrella sampling. Key features include advanced 3D visualization of large biomolecular systems, interactive editing, flexible atom selection, system pruning for efficient QC/MM setup, orbital and electrostatic potential surfaces, automated log parsing, and trajectory analysis. EasyHybrid integrates these tools into a single platform, offering a familiar yet specialized environment for quantum chemistry and hybrid QC/MM simulations.

## Graphical abstract

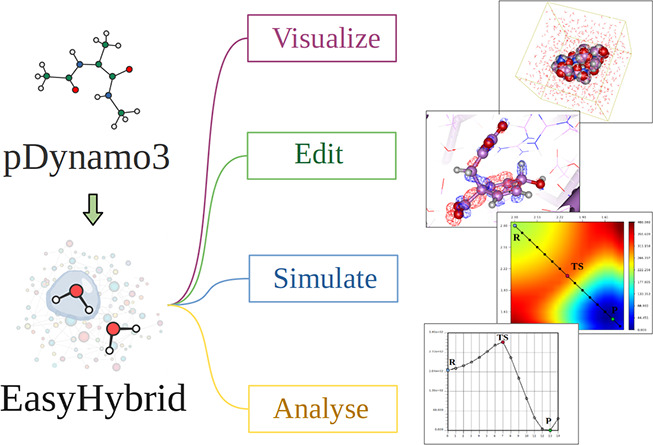

## Introduction

Interactive graphical interfaces play a crucial role in the field of computational chemistry and molecular modeling. Far beyond functioning as simple visualizers, these tools provide essential capabilities such as molecular drawing and editing, interconversion between file types and formats, and the generation and submission of simulation input files. In this context, a variety of graphical tools have been developed to address the different needs within this area of research. Notable examples include wXMacMolPlt,^1^ ECCE,^2^ and GaussView,^3^ which are interfaces specifically designed to support the quantum chemistry software packages GAMESS-US,^4^ NWChem,^5^ and Gaussian,^3^ respectively.

In contrast, programs such as Avogadro^6^ and Gabedit^7^ are general-purpose molecular editors that support multiple computational chemistry packages simultaneously. Molden^8^ represents another widely used graphical interface with broad compatibility. Despite its somewhat outdated visual appearance, it remains popular due to its excellent support for visualizing computational results, such as those generated by Gaussian. These tools are often used in combination and provide solid support for molecular drawing and editing, input generation, and output visualization. However, their primary focus is on relatively small molecular systems, typically limited to a few hundred atoms.

The limitations of these tools are not necessarily related to performance degradation, although this can occur, but rather to the lack of adequate visualization and manipulation features for larger systems. In such cases, alternatives such as PyMOL,^9^ Chimera,^10,11^ and VMD^12^ have proven effective. Generally, the first two are geared toward structural biology applications, including the visualization of proteins and other biomolecular structures, while the latter is commonly used for viewing data generated from molecular dynamics simulations. Despite their distinct design goals, all three exhibit excellent performance when handling systems comprising tens or even hundreds of thousands of atoms and offer strong usability along with sophisticated visualization capabilities. Nonetheless, they are not particularly efficient as molecular editors and provide limited support for quantum chemistry data visualization.

These challenges become especially pronounced when dealing with hybrid QC/MM (or QM/MM) systems. In such cases, a large number of atoms are described using a classical force field, such as AMBER^13^ or CHARMM,^14^ while a smaller, chemically active region is treated with quantum chemical methods. To the best of our knowledge, there is currently no free and open-source interactive environment specifically designed to facilitate the editing, manipulation, execution, and analysis of such systems. Commercial tools, such as Schrödinger’s Maestro in conjunction with the QSite module,^15^ do provide integrated support for setting up, executing, and visualizing QC/MM simulations; however, their costs are prohibitive for many researchers and institutions. Likewise, web-based interfaces such as CHARMM-GUI^16^ are limited to assisting in the preparation of QC/MM input files and structural visualization during system setup. Against this background, EasyHybrid stands out by offering an accessible, open-source, and fully integrated platform specifically designed for the pDynamo3 ecosystem, providing an entry point for the academic community seeking advanced methodologies for free. It was in this context that we originally developed GTKDynamo^17^ (no longer supported), a Python 2 plugin for the widely used PyMOL viewer, designed to support the pDynamo^18^ simulation and modeling library, versions 1.7 and 1.9. Recently, the pDynamo library was ported to Python 3 ^19^ and released under the name pDynamo3,^20^ featuring substantial rewrites and functional expansions that provide users with a broader set of capabilities. As in previous versions, the primary focus of pDynamo3 remains the application of QC/MM methodologies, although it is not limited to them. One of the key advantages of this library is the flexibility and high degree of customization afforded to simulation workflows, as its input files are essentially Python scripts that invoke the desired routines. This design allows virtually unlimited customization possibilities. However, it also introduces a steep learning curve for new users, requiring familiarity with Python programming and almost exclusively relying on a command-line interface. Moreover, system visualization is performed indirectly, often necessitating the export of coordinate files to external applications such as PyMOL or VMD, which is neither efficient nor user-friendly.

## Interface Overview and Implementation

The EasyHybrid interface is implemented in Python3 and utilizes the GTK3 toolkit for the generation of graphical windows. Its interactive 3D visualization area functions as a GTK3 widget, developed in a Python 3 module named VISMOL, which is distributed alongside EasyHybrid but maintained as a parallel project by the same development team. This modular design enables easy integration of VISMOL into GTK3 container applications, offering a flexible solution for developers seeking to embed molecular 3D visualization capabilities into their own tools. VISMOL leverages modern OpenGL (version 3.6), incorporating geometry shaders in addition to the more widely used fragment and vertex shaders. This results in significant performance gains for specific rendering modes, particularly the ’line’ and ’stick’ representations. While the technical details of VISMOL’s rendering strategies are beyond the scope of this article, further information and source code are available in the official GitHub repository.^21^

The main EasyHybrid window integrates six key components: a menu bar for all interface functionalities, a toolbar with shortcuts to common operations, a side treeview listing loaded systems and visual objects, a bottom panel with action logs and a residue viewer, a status bar summarizing system properties, and a central interactive 3D canvas. An overview of the interface is shown in Figure 1. Additional information is available at the official support page.^22^

**Figure 1:**
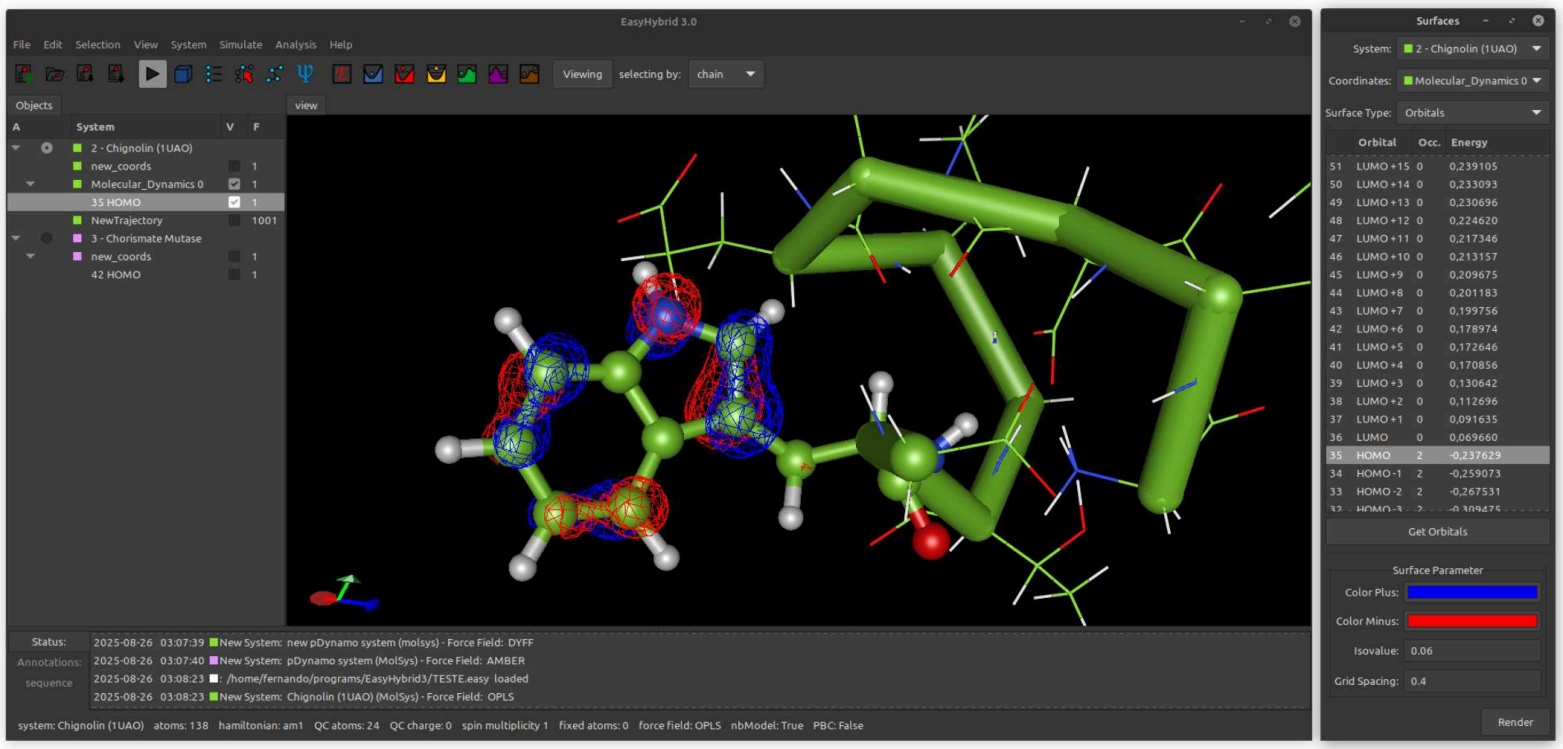
Overview of the EasyHybrid interface. The figure shows a hybrid QC/MM system, where the MM region is represented by lines and the QC region by ball-and-stick. The peptide backbone is highlighted using a thick stick representation (Cα trace), and the blue and red mesh depicts the highest occupied molecular orbital (HOMO).

EasyHybrid allows users to manage and manipulate multiple systems simultaneously within a single session. Upon loading a new system, it is automatically added to the main treeview and assigned a unique header and color code. These color codes are, by default, applied to the carbon atoms of visualizable objects, facilitating rapid identification of which atoms belong to which systems. Users can switch between active systems via radio buttons in the treeview, enabling targeted edits and simulations. Displayable objects may originate from simulation outputs or be imported from external coordinate files. These objects are dynamic: newly imported coordinate sets can either update existing displayable objects or be used to generate new ones, allowing users to aggregate different trajectories within a single object. Frame navigation is supported via the trajectory panel or arrow keys, and multiple objects can be displayed simultaneously or toggled independently, providing flexibility in comparing structural changes before and after simulations. The interactive 3D viewer was designed to efficiently render systems containing tens of thousands of atoms without noticeable performance degradation, making the software well-suited for handling complex molecular systems. Mouse interaction paradigms for rotation, centering, and selection are modeled after widely adopted practices in tools such as PyMOL and Coot,^23^ offering a familiar experience to users of those platforms. Additional user-friendly features include scroll-based zoom control critical when navigating large molecular systems—and contextual right-click menus within the 3D canvas. These menus provide streamlined access to advanced options, such as defining quantum regions for QC calculations or adjusting the visual representation of specific parts of the system.

## Selection Modes

EasyHybrid employs two distinct types of atom selection mechanisms: viewing selections and picking selections, both of which are carried out interactively via mouse clicks on atoms of interest. The selection mechanism follows a *click-on*/*click-off* logic, meaning that the same action used to select atoms is also used to deselect them. Examples of viewing and picking selections are shown in Figure S1.

Viewing selections are indicated by cyan-colored dots (the color can be changed by the user if desired). There is no specific order in which atoms of interest are selected, and the total number of atoms that can be selected is arbitrary. This type of selection is designed for a variety of tasks, including freezing atom positions, changing atom colors, defining quantum mechanical (QM) regions, pruning or deleting parts of the system, among others. Selections are dynamic and support both additive and subtractive modifications, allowing users to update them incrementally. EasyHybrid also enables saving and reusing these selections in later simulation steps. The selection criteria available in the viewing selection mode include:

- **By Atom**: Selects only the clicked atom.
- **By Residue**: Selects all atoms belonging to the residue of the clicked atom.
- **By Chain**: Selects all atoms belonging to the same chain.
- **By C-Alpha**: Selects only the clicked C-alpha atom.
- **By Molecule**: Selects the entire molecule containing the clicked atom.
- **By Solvent**: Selects atoms associated with solvent molecules (e.g., water).

The alternative mode, picking selection, follows a different paradigm. Here, up to four atoms can be selected, and the order in which they are picked is strictly preserved. This mode is particularly useful for operations that require atom ordering, such as calculating distances, bond angles, and dihedral angles, or for defining reaction coordinates. In the 3D visualization, selected atoms are represented as colored spheres, each labeled with a tag indicating its selection order, as illustrated in item b, Figure S1.

## Representation Types

EasyHybrid is designed to work with systems containing large numbers of atoms, particularly biological systems. To enhance user experience, it provides various molecular structure representations. Currently, the available representation types include: wireframe, sticks, sticks with dynamic bonds, atomic spheres, van der Waals spheres, ribbons or C-alpha trace, and wireframe for nonbonded atoms (Figure S2). Once activated through prior selection, representations are applied to all frames of a trajectory associated with a given visual object. Representations follow an additive logic, meaning that users can add multiple representation segments in successive events, or remove them as needed. The same logic applies to color assignments: by default, carbon atoms are shown using the reference color associated with the system, but users can select specific parts of the model and assign different colors as desired. Since many molecular trajectories involve atom transfer (i.e., bond formation and breaking), EasyHybrid includes a Dynamic Bonds representation. Visually similar to the sticks representation, Dynamic Bonds update dynamically across trajectory frames. Unlike other representations, this mode is not additive: the user must provide the full selection of atoms to be rendered this way at once. By default, bonds within the quantum region are shown using Dynamic Bonds. As in pDynamo3, the criteria for bond formation and breaking are based on the sum of the atoms’ covalent radii multiplied by a correction factor, which can be adjusted. Additionally, EasyHybrid supports surface representations for molecular orbitals, electron densities, and electrostatic potential maps. These surfaces can be linked to trajectories, allowing visualization of changes such as the evolution of frontier orbitals during a molecular dynamics simulation. Figure S3 shows the HOMO representation for a potential energy surface scan of the reaction coordinate in chorismate mutase.

## File Types and System Preparation

EasyHybrid can read and export pDynamo3 serialization files in.pkl and.yaml formats, providing flexibility for simulation setup and execution outside of the GUI. These files contain all system information, including coordinates and QC/MM parameters. Upon loading, EasyHybrid displays MM atoms as lines, QC atoms as ball-and-stick (dynamic), and fixed atoms in gray, facilitating system inspection. Coordinates can also be exported to commonly used formats in computational chemistry, such as .xyz, .pdb, .mol, and .mol2. Entire sessions can be saved using EasyHybrid’s native project file format.easy, which compiles all loaded systems and configuration settings into a single file. The most direct method to load a system into EasyHybrid is via a coordinate file. However, to perform any simulation, the system must be associated with an energy model. The simplest model is pure quantum chemistry, as coordinates are often sufficient. However, QC-only simulations are suitable only for small systems due to their high computational cost.

EasyHybrid provides a dedicated window for setting up QC calculations (see Supplementary Figure). Users can choose between native pDynamo3 methods or external software such as ORCA,^24^ xTB,^25^ and DFTB+,^26^ all of which interface with pDynamo3. Each of these options includes dedicated auxiliary windows for setting the required parameters. Associating a system with a molecular mechanics (MM) model is more complex, as it requires topological information in addition to atom types and coordinates. MM systems can be built using force fields natively supported in pDynamo3 (e.g., OPLS, CHARMM, DYFF, pDynamo3’s version of the Universal Force Field^27^). In this case, users must provide a file with topological information (e.g., .mol2) and a compatible parameter set. The interface suggests default parameter files, but users may substitute their own if desired.

Alternatively, MM systems prepared in external packages such as Amber (.top, .crd) or CHARMM (.chm, .psf, .par) can be imported and manipulated in EasyHybrid. Several operations rely on viewing selections. Two critical features include atom fixation and system pruning, both of which can significantly reduce computational cost:

- **Atom Fixation**: freezes selected atoms during simulations (e.g., geometry optimization or molecular dynamics), preventing them from moving.
- **Pruning**: creates a reduced-size system based on selected atoms.

A third important use of atom selection is in defining the quantum region in QC/MM hybrid systems. In this case, users must begin with an MM-defined system and then select atoms to designate as part of the QC region. EasyHybrid automatically adjusts the partial charges in the remaining MM portion of residues that are partially included in the QC region. This adjustment is performed by redistributing the residual charge required to reach the nearest integer value, dividing it into equal fractions, and assigning these fractions to the remaining atoms in the MM portion of the residue. This procedure ensures that the total MM charge remains an integer, as required by most QC/MM methods. The program also stores the original charges, so that when a new quantum region is defined, EasyHybrid initially restores the original charges, minimizing the possible accumulation of errors. Figure 2 illustrates the steps for defining the QC region in EasyHybrid.

**Figure 2:**
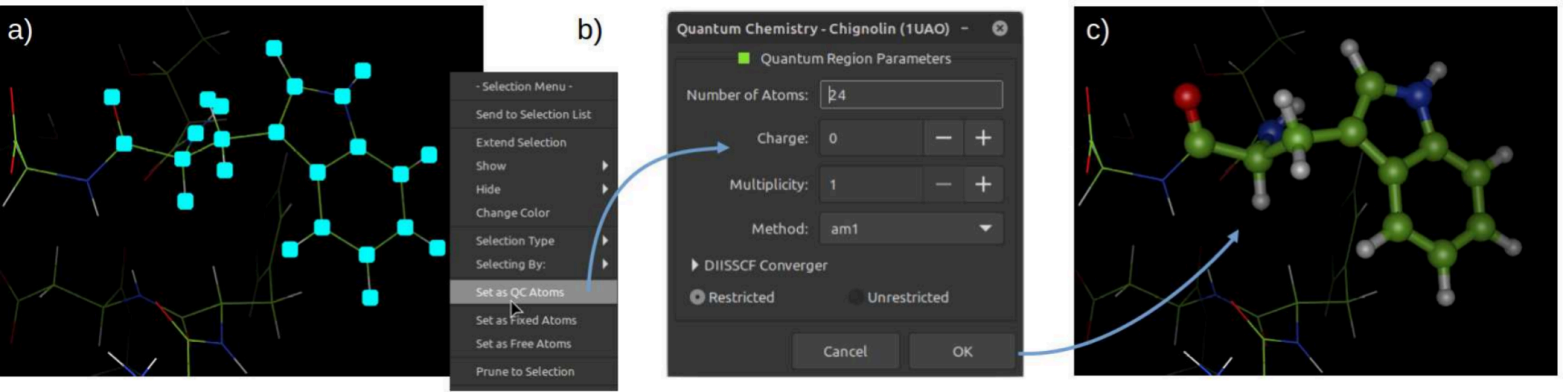
QC region selection and setup in EasyHybrid: (a) atom selection in viewing mode with access to the Quantum Chemistry Setup window via the right-click menu; (b) configuration of QC parameters; (c) default representation of QC atoms as ball-and-stick and MM atoms as lines.

## Simulation Types

EasyHybrid provides a comprehensive suite of simulation tools covering a wide range of computational chemistry protocols, compatible with quantum mechanical (QC), molecular mechanical (MM), and hybrid QC/MM potential models. The available simulation types include:

- **Single-Point Energy Calculations**: Energy evaluations can be performed using MM, QC, or hybrid QC/MM potentials. These calculations are useful for benchmarking, comparing system configurations, or preparing structures for further simulations.
- **Geometry Optimization**: Structure minimization is supported using the steepest descent and conjugate gradient algorithms implemented in the pDynamo3 library. The user can specify the number of optimization cycles, convergence criteria, and whether or not to save the trajectory of intermediate structures during the optimization process.
- **Molecular Dynamics (MD) Simulations**: EasyHybrid supports classical MD simulations using three integrators: velocity Verlet, leapfrog, and stochastic Langevin dynamics. The interface allows the definition of standard simulation parameters such as time step, total simulation time, temperature, pressure, trajectory saving frequency, and random seed.
- **Nudged Elastic Band (NEB) Reaction Path Calculations**: NEB^28^ is used to identify minimum energy reaction paths between two end point structures (typically reactant and product). The interface enables the user to define the initial and final geometries, the number of intermediate images (or replicas), and other NEB-specific parameters. These images are then optimized to locate the minimum energy path on the potential energy surface (PES).
- **Potential Energy Surface (PES) Scans**: One-dimensional (1D) and two-dimensional (2D) PES scans can be performed by applying geometric constraints—typically distance restraints or linear combinations thereof. At each scan point, a geometry optimization is conducted. The user can define the coordinate range, number of scan points per dimension, and the force constant associated with the restraint.
- **Potential of Mean Force (PMF) Simulations via Umbrella Sampling**: PMF calculations provide free energy profiles as a function of a chosen reaction coordinate. The implementation in EasyHybrid closely mirrors that of PES scans but uses short MD simulations at each window instead of geometry optimizations. The reaction coordinate trajectory obtained from each window can be postprocessed using the Weighted Histogram Analysis Method (WHAM),^29,30^ as implemented in pDynamo3, to reconstruct the overall free energy surface.

All simulation types are executed via pDynamo3′s backend and benefit from EasyHybrid’s integrated visualization, selection, and configuration tools. For QC and QC/MM simulations, users can employ either native pDynamo3 methods or pDynamo3 combined with external engines (e.g., ORCA, xTB, DFTB+), all accessible through dedicated interface panels.

## Results and Analysis

Simulations executed using the pDynamo3 library generate results in a variety of formats. In EasyHybrid, all pDynamo3 processes are designed to output a log file that contains the essential results of a given simulation. EasyHybrid can automatically read and interpret these log files, displaying relevant data in graphical form. These plots can be saved and manipulated by the user, providing a convenient means of producing both graphical and structural representations.

Log file processing is triggered automatically at the end of any pDynamo3 routine executed through EasyHybrid, but it can also be performed manually for previously generated EasyHybrid/pDynamo3 log files. Plotting is handled by a custom-built tool named EasyPlot, developed using the Pycairo graphics library. Figure 3 presents a visual analysis of a simultaneous scan along two reaction coordinates, plotted with EasyPlot. Another important class of output files produced by pDynamo3 includes trajectory files. These may come in various formats, both native (e.g., pkl) and external (e.g., CRD, NetCDF, and DCD), and may contain atomic coordinates, energies, reaction coordinate values, velocities, and more. EasyHybrid supports multiple pDynamo3 trajectory types, allowing users to load several trajectories simultaneously and specify which data objects to process. The interface also features a set of structural analysis tools, including the monitoring of multiple distances, angles, or dihedrals over the course of a trajectory, as well as calculations, such as RMSD, structural alignment, reimaging, among others.

**Figure 3:**
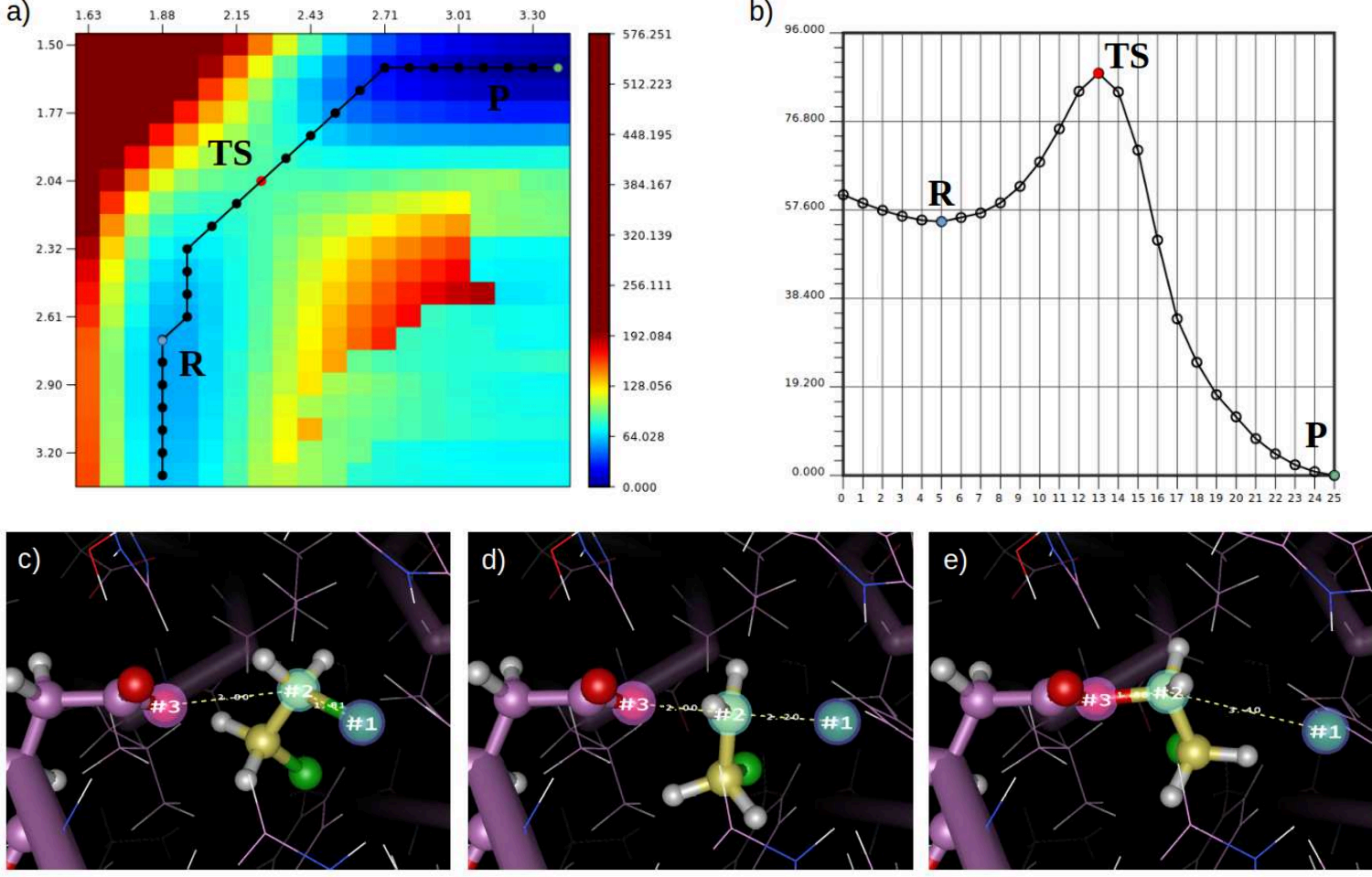
Potential energy surface (PES) scan along two reaction coordinates performed simultaneously. (a) Energy matrix plot, where the horizontal (*x*) and vertical (*y*) axes correspond to reaction coordinates r1 and r2, respectively. Labels for reactants (R), transition state (TS), and products (P) were added later during figure composition. The user can interactively select frames from the energy surface to generate (b) a one-dimensional energy profile, facilitating analysis as a reaction trajectory. Alternative reaction paths can be analyzed by selecting a different sequence of frames, such as the apparent pathway through the lower right corner of the PES (which, in this case, is an artifact). Representative structures are shown for (c) reactants, (d) transition state, and (e) products. Markers 1, 2, and 3, centered on semitransparent spheres, indicate the selected atoms (picking mode) that define the reaction coordinates, while dashed lines show the interatomic distances dynamically tracked along the trajectory. Energies were calculated using the hybrid AMBER ff99S/PM6 model. All plots were generated with EasyPlot.

## Comparison with GTKDynamo and Other Molecular Editors

EasyHybrid represents a major evolution of the earlier GTKDynamo project, which was originally designed to support pDynamo versions 1.7 to 1.9 (Python 2-based). In GTKDynamo, the user interface combined GTK2-based windows with PyMOL 1.8 as the 3D viewer, and Matplotlib was used for plotting. In contrast, EasyHybrid has been updated to utilize modern versions of Python and GTK. Notably, PyMOL has been replaced by a custom 3D rendering engine built with modern OpenGL/GLSL, and Matplotlib has been entirely replaced by the in-house EasyPlot module for graphical visualization. The user experience and interface design of EasyHybrid has been influenced by several leading molecular visualization programs, including PyMOL, VMD, Avogadro, wXMacMolPlt, and Gabedit. The authors acknowledge the substantial contributions of these projects and their long-standing impact on the community. However, for the purposes outlined in this work, EasyHybrid is unique in providing a user experience specifically optimized for managing pDynamo3-based QC/MM systems. Table S1 shows some features implemented in EasyHybrid in comparison with other well-established free software in the area of computational chemistry.

## Summary

In this work, we have presented EasyHybrid, a modern, free, and open-source graphical environment specifically designed to facilitate quantum/classical hybrid simulations. Built to complement the pDynamo3 simulation library, EasyHybrid provides a cohesive platform for molecular system preparation, simulation control, trajectory analysis, and advanced visualization. By combining the flexibility of scripting in Python with an interactive and user-friendly interface, EasyHybrid facilitates access for both students beginning to explore computational chemistry and hybrid methodologies, and experienced researchers who seek to accelerate the preparation and analysis of complex simulations. Its ability to handle large systems, define quantum regions intuitively, and support advanced protocols such as NEB and umbrella sampling makes it a valuable tool for computational chemists and structural biologists alike. EasyHybrid is freely available for Linux under the GNU General Public License and can also be run on Windows via the Ubuntu terminal (WSL) or on Windows and macOS through a virtual machine. Additional information can be found on our project homepage.

## Supplementary Material



## Data Availability

EasyHybrid’s detailed documentation, examples, tutorials, and installation instructions are available at https://sites.google.com/view/easyhybrid and https://www.youtube.com/@EasyHybrid. EasyHybrid’s source code is available at https://github.com/ferbachega/EasyHybrid3. The official VISMOL GitHub repository is available at https://github.com/casebor/Vismol/tree/vismol_easyhybrid.

## Notes

Figures S1–S3 and Table S1: Additional materials illustrating EasyHybrid. S1: Selection types (viewing and picking selections with visual tags). S2: Representation types (lines, sticks, ball-and-stick, ribbons and van der Waals spheres). S3: Molecular orbital (HOMO) along a chorismate mutase reaction path and potential energy landscape. Table S1: Comparison of EasyHybrid with other molecular visualization programs (PDF)
