# Supplementary material for: EasyHybrid: An Interactive Graphical Environment for Quantum, Classical and Hybrid Simulations with pDynamo3
Source: J Chem Inf Model. 2025 Nov 11;66(3):1286–92. doi: 10.1021/acs.jcim.5c02047 (PMC12892311; doi:10.1021/acs.jcim.5c02047)
Supplement: Supplementary file 1 [file ci5c02047_si_001.pdf]

EasyHybrid: An Interactive Graphical Environment for Quantum, Classical and Hybrid Simulations with pDynamo3

Jose Fernando R. Bachega [*,†,‡] Gustavo Hagen [‡] Carlos Sequeiros-Borja [¶]
Kai Nikklas [§] Jorge Chahine [‖] Luis Fernando M. S. Timmers [⊥] and Martin J. Field [*,#]

†*Department of Pharmacosciences, Federal University of Health Sciences of Porto Alegre, 90050-170 Porto Alegre – RS, Brazil*

‡*Biotechnology Center, Graduate Program in Molecular and Cellular Biology, Federal University of Rio Grande do Sul, 90650-001 Porto Alegre , RS, Brazil*

¶*Tunneling Group, Biotechnology Center, Krzywoustego 8, 44-100 Gliwice, Poland*

§*3D Development, deCode GmbH, 46145 Oberhausen, Germany*

‖*Biological Structures Group, Multiuser Center for Biomolecular Innovation, São Paulo State University, São José do Rio Preto 15054-000, SP, Brazil*

⊥*Graduate Program in Biotechnology, University of Vale do Taquari, 95900-000 Lajeado, RS, Brazil*

#*Laboratoire de Chimie et Biologie des Métaux, UMR5249, Université Grenoble I, CEA, CNRS, 38054 Grenoble, France; Theory Group, Institut Laue-Langevin, 38042 Grenoble, France*

E-mail: easyhybrid3@gmail.com; field@ill.fr

Phone: +55 (51) 3303-8801

Table S1: A comparison between EasyHybrid and other molecular visualization programs.

**Program / Feature**	**Handling of Large Atomistic Systems**	**Dynamic Bonding & Reaction-Focused Representations**	**Quantum Calculation Input and Data Processing**	**QC/MM Support**
**PyMOL**	Can handle systems with hundreds of thousands of atoms and thousands of frames	Offers various representations focused on biological molecules, but lacks dynamic bonding representation	No specific support, but can read `.cube` volume files	Not supported
**VMD**	Can handle systems with hundreds of thousands of atoms and thousands of frames	Offers various representations focused on biological molecules, including dynamic bonding representation	No specific support, but can read `.cube` volume files	Not supported
**GABEDIT**	Limited to small systems, with significant performance and usability loss on larger ones	Basic representations only, but includes dynamic bonding representation	Supports many quantum chemistry programs and includes its own graph plotting tool	Not supported
**Avogadro**	Reduced performance and limited visualization features for large systems	Offers advanced representations such as ribbons and cartoons, including dynamic bonding representation	Supports some quantum chemistry and molecular mechanics programs, but limited support for result analysis	Not supported
**EasyHybrid**	Can handle systems with hundreds of thousands of atoms and thousands of frames	Offers advanced representations such as ribbons and cartoons, including dynamic bonding representation	Supports main pDynamo simulation routines, whether pure QC, MM, or QC/MM, including advanced sampling techniques like umbrella sampling	Full support for QC/MM systems, including region definition and analysis

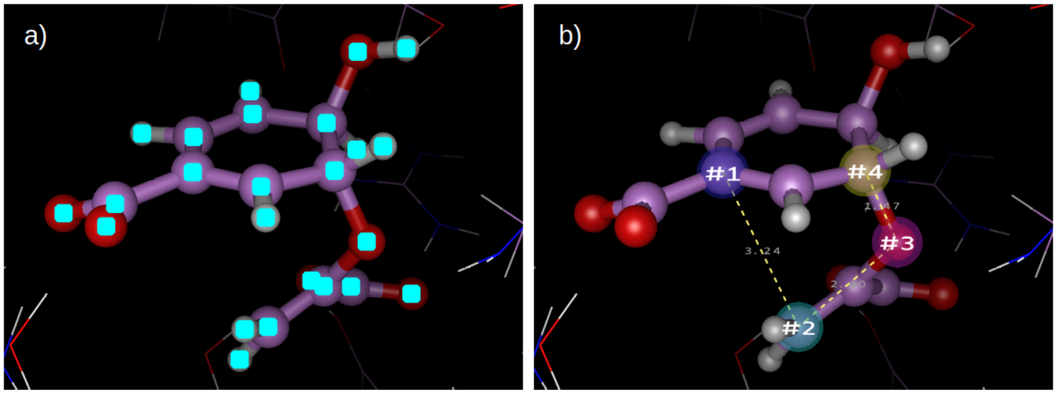

Figure S1: Selection types: (a) viewing selection; (b) picking selection showing four atoms selected in sequence with visual tags indicating their selection order

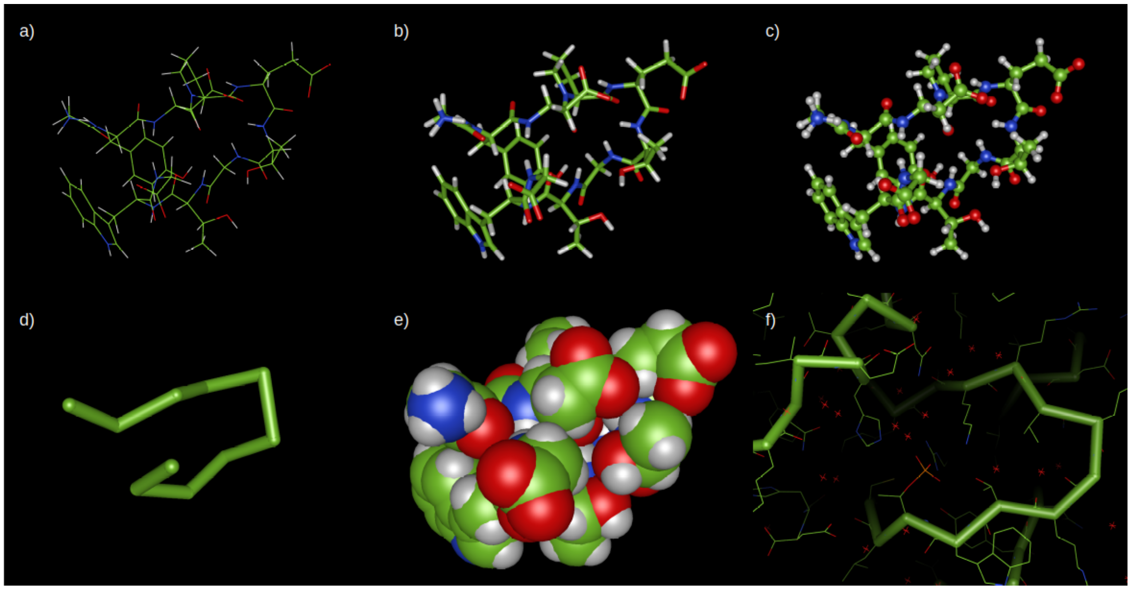

Figure S2: Representation types available in EasyHybrid: (a) lines (wireframe); (b) sticks; (c) atomic spheres with sticks (ball and sticks); (d) ribbons (Cα trace); and (e) van der Waals spheres, and, f) Combination of ribbons, lines and nonbonded lines.

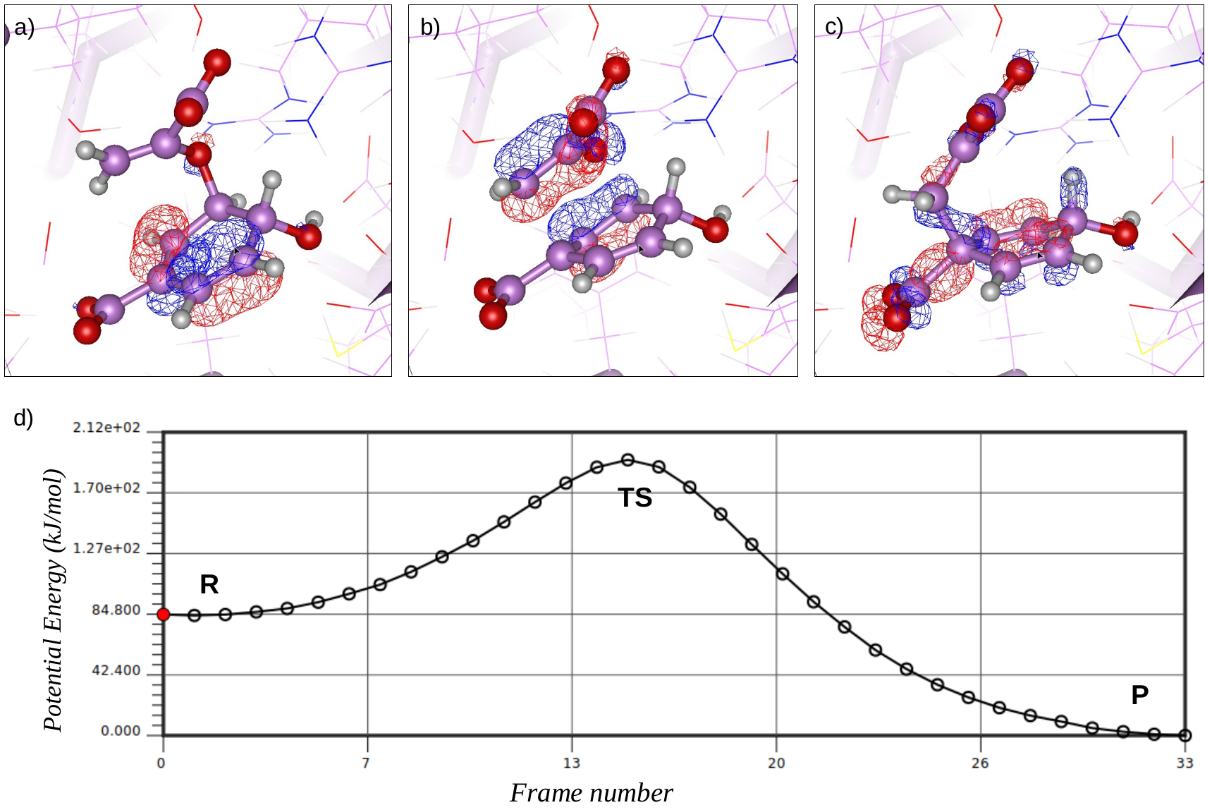

Figure S3: EasyHybrid molecular orbital (HOMO) representation of a reaction path calculation in chorismate mutase: **(a)** reactants, **(b)** transition state, and **(c)** products. The QM region is shown in ball-and-stick representation. **(d)** Potential energy landscape along the reaction coordinate. A tutorial to reproduce this calculation is available on the EasyHybrid website.

Data and Software Availability

EasyHybrid's detailed documentation, examples, tutorials, and installation instructions are available at `https://sites.google.com/view/easyhybrid` and `https://www.youtube.com/@EasyHybrid`

EasyHybrid's source code is available at `https://github.com/ferbachega/EasyHybrid3`

The official VISMOL GitHub repository is available at `https://github.com/casebor/Vismol/tree/vismol_easyhybrid`.

Author Contributions

J.F.R.B. conceived and developed EasyHybrid and co-authored the 3D graphics module Vismol. **G.H.** contributed to code implementation and manuscript writing. **C.S.B.** served as the main developer of the Vismol library. **K.N.** provided expertise in OpenGL and developed shader code for Vismol. **J.C.** tested the software and suggested improvements to the interface. **L.F.M.S.T.** co-created the EasyHybrid project and contributed to interface design. **M.J.F.** created pDynamo3 and contributed to the EasyHybrid–pDynamo3 interface. All authors contributed to the manuscript.

Conflict of Interest

The authors declare no competing financial interests.
